# Supplementary figures and images for: Long-read transcriptomics of caviid gammaherpesvirus 1: compiling a comprehensive RNA atlas
Source: mSystems. 2025 Feb 27;10(3):e01678-24. doi: 10.1128/msystems.01678-24 (PMC11915868; doi:10.1128/msystems.01678-24)

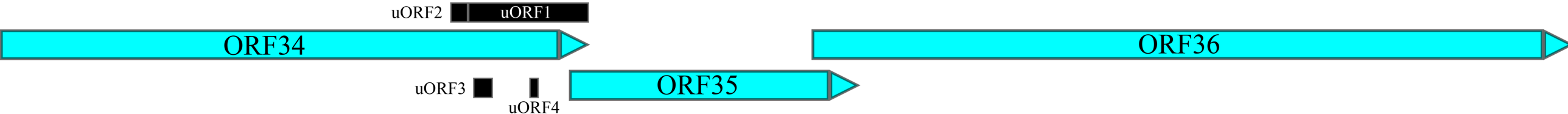

**Supplemental Figure 1. Upstream ORFs in the ORF35 transcript**

Supplement: Figure S1 — Upstream ORFs in the ORF35 transcript. [file msystems.01678-24-s0001.pdf]
